# The Association of Outdoor Activity and Age-Related Cataract in a Rural Population of Taizhou Eye Study: Phase 1 Report

**DOI:** 10.1371/journal.pone.0135870

**Published:** 2015-08-18

**Authors:** Yating Tang, Yinghong Ji, Xiaofang Ye, Xiaofeng Wang, Lei Cai, Jianming Xu, Yi Lu

**Affiliations:** 1 Department of Ophthalmology, Eye and ENT Hospital of Fudan University, 83 Fenyang Road, Xuhui District, Shanghai, China; 2 Myopia Key Laboratory of the Health Ministry & Visual Impairment and Reconstruction Key Laboratory of Shanghai, Shanghai, China; 3 Fudan University and Shanghai Key Laboratory of Meteorology and Health, Pudong Meteorological Service, Shanghai, China; 4 State Key Laboratory of Genetic Engineering and MOE Key Laboratory of Contemporary Anthropology, Collaborative Innovation Center for Genetics and Development, School of Life Sciences and Institutes of Biomedical Sciences, Fudan University, Shanghai, China; 5 Fudan-Taizhou Institute of Health Sciences, 1 Yaocheng Road, Taizhou, Jiangsu Province, China; 6 Shanghai Key Laboratory of Meteorology and Health, Pudong Meteorological Service, Shanghai, China; Sun Yat-sen University, CHINA

## Abstract

**Purpose:**

To study the relationship between outdoor activity and risk of age-related cataract (ARC) in a rural population of Taizhou Eye Study (phrase 1 report).

**Method:**

A population-based, cross-sectional study of 2006 eligible rural adults (≥45 years old) from Taizhou Eye Study was conducted from Jul. to Sep. 2012. Participants underwent detailed ophthalmologic examinations including uncorrected visual acuity (UCVA), best corrected visual acuity (BCVA), intraocular pressure (IOP), slit lamp and fundus examinations as well as questionnaires about previous outdoor activity and sunlight protection methods. ARC was recorded by LOCSⅢ classification system. The prevalence of cortical, nuclear and posterior subcapsular cataract were assessed separately for the risk factors and its association with outdoor activity.

**Results:**

Of all 2006 eligible participants, 883 (44.0%) adults were diagnosed with ARC. The prevalence rates of cortical, nuclear and posterior subcapsular cataract per person were 41.4%, 30.4% and 1.5%, respectively. Women had a higher tendency of nuclear and cortical cataract than men (OR = 1.559, 95% CI 1.204–2.019 and OR = 1.862, 95% CI 1.456–2.380, respectively). Adults with high myopia had a higher prevalence of nuclear cataract than adults without that (OR = 2.528, 95% CI 1.055–6.062). Multivariable logistic regression revealed that age was risk factor of nuclear (OR = 1.190, 95% CI 1.167–1.213) and cortical (OR = 1.203, 95% CI 1.181–1.226) cataract; eyes with fundus diseases was risk factor of posterior subcapsular cataract (OR = 6.529, 95% CI 2.512–16.970). Outdoor activity was an independent risk factor of cortical cataract (OR = 1.043, 95% CI 1.004–1.083). The risk of cortical cataract increased 4.3% (95% CI 0.4%-8.3%) when outdoor activity time increased every one hour. Furthermore, the risk of cortical cataract increased 1.1% (95% CI 0.1%-2.0%) when cumulative UV-B exposure time increased every one year.

**Conclusion:**

Outdoor activity was an independent risk factor for cortical cataract, but was not risk factor for nuclear and posterior subcapsular cataract. The risk of cortical cataract increased 4.3% when outdoor activity time increased every one hour. In addition, the risk of cortical cataract increased 1.1% (95% CI 0.1%-2.0%) when cumulative UV-B exposure time increased every one year.

## Introduction

Age-related cataract (ARC) remains the predominant cause of blindness all over the world, especially in China, home to 1/5 of the world’s population[1,2]. The Beijing Eye Study showed that the prevalence of cataract was 53.1% in adults ≥40 years old in China[3]. Cataract surgery is the only effective treatment of cataract and still expensive in developing countries at present. With the increasing aging population, the morbidity and burden of ARC is expected to increase[4,5], which means heavier load to public health care. Therefore, it is very important to study the risk factors and mechanisms of cataract and to carry out some prevention work by epidemiologic method. However, so far in mainland China, most of the epidemiologic studies stress more on the prevalence of ARC and cataract surgery[3,6,7,8,9,10]. Population-based epidemiologic studies focusing on risk factors of ARC in China are still rare when compared to Unite States[11,12,13], Australia[14], Singapore[15,16] and Europe[17,18].

Although age is the most important risk factor of ARC, there are some other potential risk factors that may influence the development of ARC including gender, smoking, sunlight exposure, diabetes and drug intake[19]. However, the risk factors vary from different countries and living environment. For example, sunlight exposure was proved to be significantly related to cataract in some European studies[20,21], but no correlation was found in Beaver Dam Eye Study[22]. Furthermore, the relationship between sunlight exposure and risk factor of cataract has been seldom studied in mainland China. Therefore, we studied the association of outdoor activity with risk factor of cortical, nuclear and posterior subcapsular cataract in a rural population of Taizhou Eye Study, China. This report is an exploration of the relationship between outdoor activity as well as some other risk factors and ARC development.

## Methods

### Study Population

The population and data for this study were derived from the Taizhou Eye Study. The details of the population and methods of Taizhou Eye Study have been previously described[23]. In brief, Taizhou Eye Study is part of a large scaled prospective study called Taizhou Longitudinal Study carried out from 2007[24]. For Taizhou Eye Study, it is an ongoing large-scale population based on prospective cohort study carried out from April 2012 in Taizhou City, Jiangsu Province, middle-east part of China. Taizhou Eyes Study focuses primarily on the prevalence, incidence and risk factors of ARC and other age-related eye diseases. The total population of this study was about 10,000 adults aged ≥45 years old. In this report, we randomly selected 4 villages with a rural population of 2600 adults aged ≥45 years old. Of these individuals, 2006 eligible participants (77.2% response rate) finished the total procedure from Jul. to Sep. 2012. Within one week prior to the baseline survey, related advertisement material were distributed to every household of the aimed community by the study group. All participants were self-identified Han Chinese (at least four generations were Han Chinese) and local residents for at least 10 years. This study adhered to the Declaration of Helsinki and was approved by the Human Ethics Committee of School of Life Science of Fudan University. Consents in written form were also obtained from all participants prior to participation.

### Interview and Clinical Examination Procedure

At each examination site, participants were brought to the nearby village clinics or village offices for general physical, detailed ophthalmic examinations and questionnaires on prescheduled examination days. Those who failed to come to the examination site were revisited. Those physically disabled and those who still failed to come to the examination site after second visit were conducted an ocular examinations (using portable equipment) in their home at the end of the work in every examination site.

The detailed examination procedure has been reported[23]. In brief, after registering of name, ID, gender and age and some general examination (including heart rate, blood pressure, body mass index, etc.), vein blood was collected for serological and genetic analysis in the future. Presenting visual acuity (PVA, wearing present correction if any) was measured using a retro-illuminated logarithm E chart with the minimum angle of resolution at a distance of 4 meters and at 1 meter for those who failed to read the top line figures (20/200), as Zhao[25] et al. used in the Nine-Province Eye Survey. When PVA was ≤20/40 in either eye, the best corrected visual acuity (BCVA) was determined by subjective refraction without cycloplegia. Intraocular pressure was measurement by Icare rebound tonometry (Icare TAO1i, Helsinki, Finland). A-scan (AL-3000, TOMEY, Tokyo, Japan) was performed to measure the axial length (AL), central anterior chamber depth (ACD) and lens thickness of all participates under topical anesthesia. The examinations were repeated if the ophthalmologist considered the results unreliable. All of these ocular examinations were carried out by 4 experienced technicians from Taizhou Eye Study Team. All of the eye technicians were required to complete standardized ophthalmologic training and had certification to conduct the eye examinations. The examination consistency was over 95% between different examiners.

Lens and ocular anterior segment examination were evaluated using a slit-lamp (Topcon SL-8Z; Topcon, Inc., Tokyo, Japan) after dilation of the pupil to give the lens opacification records of all residents. Fundus examination was carried out by +90 diopter (D) lens at X16 magnification or direct ophthalmoscopy. Those who were under high risk of angle closure glaucoma were examined only under small pupil situation. The lens and fundus examination were carried out by one experienced ophthalmologist (Dr. Yating Tang) to make sure the consistence of the results. Individuals with typical fundus diseases were taken fundus photos after dilation of the pupil using Canon retinal camera system (CX1, Canon, Inc., Tokyo, Japan) by one experienced ophthalmologist.

The face-to-face questionnaires interviews concerning economic situation, education level, life habit, tobacco, alcohol and tea intake, activity hours outdoors, drug intake history and disease history and so on were administered by the study clerks using computers. Average daily outdoor activity hours and some sunlight protection methods (sunglass, sun hat, sun umbrella or without any protection) were recorded to evaluated the sunlight exposure condition. For quality control, the method of sampling, questionnaire design, training, physical examination, laboratory examinations, and data management have been centralized and standardized[24]. General examinations and interviewer staff were required to complete standardized training and to get certifications to conduct specific survey. All of the interviews were tape-recorded, and about 5% of the tapes were used for interviewing quality evaluation. All examination and questionnaires data were put into computer database on examination day. Computers were used to check the reasonable responses throughout the whole examination process to identify contradictory responses. Phase summary and statistical analysis was made to make sure the data was accurate, consistent and standardized.

### Grading of Cataract and Lens Opacities

Cataract were recorded using lens opacities classification system Ⅲ (LOCSⅢ)[26]. According to LOCSⅢ, nuclear lens opacities were classified into 6 grades (NO1 NC1-NO6 NC6), cortical lens opacities were classified into 5 grades (C1-C5) and posterior subcapsular cataract (PSC) 5 grades (P1-P5). A person with cataract was defined as any LOCS III grading of ≥2 in either eye and lens opacities degree was compatible with visual impairment. Cortical cataract was defined as LOCSⅢ≥2 for cortical opacities, nuclear cataract as ≥2 for nuclear opacities or ≥2 for nuclear color and subcapsular cataract as ≥2 for subcapsular opacities. Any cataract was defined as cortical, nuclear or posterior subcapsular cataract or had cataract surgery in either eye. If one eye has two or three types of cataract, for example, combined cortical and nuclear cataract, it will be classified into cortical and nuclear cataract, respectively. One individual could be classified into different groups of cataract types. Phakic status (phakic, pseudophakic or aphakic) of each eye was recorded, respectively. In eyes where lens assessement was not available, reasons were also recorded. If a participant had unilateral lens extraction, we used the LOCS III grading from the contralateral phakic eye defined the lens opacity types per person. If a participant had bilateral lens extraction (bilateral pseudophakic or aphakic eyes), we excluded him/her in specific types of cataract prevalence analysis as it was difficult to evaluate that.

### Eye Disease Diagnosis

The eye diseased diagnosis criteria have been described in our previous report[23]. Briefly, we defined myopic macular degeneration in subjects with a refractive error exceeding -6.0 dipoters, axial length of 26 mm or more, and typical degenerative myopic fundus changes[27]. We defined AMD according to the Wisconsin Age-related Maculopathy Grading System[28] and glaucoma according to the International Society for Epidemiological Ophthalmology classification[29]. Additionally, the diagnoses of diabetic retinopathy, corneal opacity, retinal detachment, pterygium, uveitis, and others diseases followed the clinical standard.

### Cumulative UV-B Exposure Years

For cumulative UV-B exposure years calculation, we used a modified and simplified formula that was derived from the Melbourne visual impairment project model[30]. The formula was shown below.

$$E=E_{c}+E_{a}=\{\begin{array}{c} Year_{c}\times \frac{H_{c}}{H}+\left(Age-Year_{c}\right)\times \frac{H_{a}}{H},\;if\;H_{a}<11h\\ Year_{c}\times \frac{H_{c}}{H}+\left(Age-Year_{c}\right),\;if\;H_{a}\geq 11h \end{array}$$

Where

E = cumulative UV-B exposure years (y).

Ec = UV-B exposure years during **children** period.

Ea = UV-B exposure years during **adult** period.

Yearsc = years of children period, that is, 18 years in total in our study.

Hc = average outdoor exposure hours during children period. For the period was school period, we defined 2 hours per day for outdoor exposure hours during this period[30].

H = maximum 11 hours (8:00–18:00) of outdoor exposure per day in Taizhou Eye Study.

Age = age of participant in Taizhou Eye Study.

Ha = average outdoor activity hours during adult period, we recorded the “Ha” using the questionnaires.

### Statistical Analysis

Statistical analysis was performed using SPSS Statistics 17.0 (IBM SPSS Inc., Chicago IL, USA). The risk factors for each type of lens opacity were calculated separately. The data are shown as mean±standard deviation form. Univariate logistic regression was used to assess the univariate association of each risk factor with cataract (cortical, nuclear and PSC vs. no cataract). Stepwise multivariable logistic regression was conducted to evaluate the independent associations for each risk indicator. The candidate risk factors include age, gender, outdoor activity time, cumulative UV-B exposure years, sunlight protection, glaucoma, age-related macular degeneration (AMD), other fundus disease (including diabetic retinopathy, macular hole, retinal detachment, branch retinal vessel occlusion, etc.). Odds ratios (OR) value and 95% confidence intervals (CI) were presented. P<0.05 was regarded as statistically significant.

## Result

Of all the 2006 eligible participants, the mean age was 60.1±9.5 years old (age range 45–100 years old). The percentage of female: male was 1189:817 (1.46:1). The mean time of ourdoor activity was 5.7±3.2 hours (time range 0–16.0 hours) and the ratio of adults who have sunlight protection measures vs those have no sunlight protection measures was 1007:994 (1.01:1).

We examined the lens conditions in 3995 eyes (right eye 1998 and left eye 1997) of 2006 people, 17 eyes (right eye 8 and left eye 9) were excluded because it was unable to evaluate the lens opacification condition with the reasons of ocular atrophy or prosthetic eye, corneal opacities, etc. In specific cataract calculation, 11 people had bilateral cataract surgery and therefore, 1995 individuals were included in the specific cataract per person analysis.

**Fig 1:**
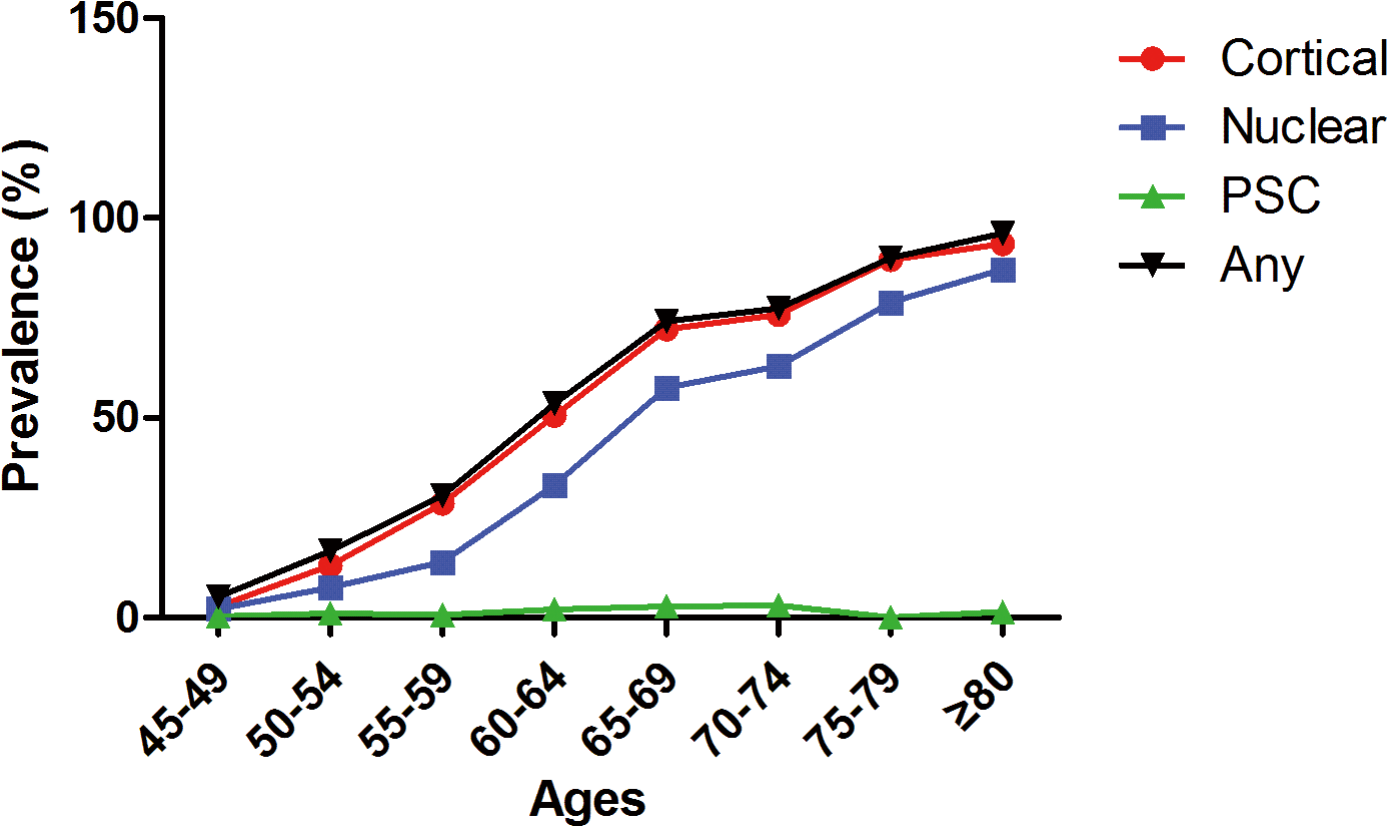
Prevalence of cortical, nuclear and any cataract per person in each age group. Ages were classified into eight continuous groups (45–49, 50–54, 55–59, 60–64, 65–69, 70–74, 75–79 and ≥80 years old). For cortical cataract, the prevalence at different age groups were 2.8%, 13.1%, 28.5%, 50.5%, 72.2%, 75.8%, 89.5% and 93.6%. For nuclear cataract, the prevalence were 2.2%, 7.6%, 13.9%, 33.2%, 57.5%, 62.9%, 78.9% and 87.2%. For posterior subcapsular cataract (PSC), the prevalence were 0.3%, 1.1%, 0.7%, 2.1%, 2.8%, 3.0%, 0.0% and 1.4%. For any cataract, the prevalence were 5.2%, 16.7%, 30.5%, 53.5%, 74.2%, 77.4%, 90.0% and 96.2%.

Table 1 showed the prevalence of cataract and cataract surgery per eye and per person in this study. There were in total 1627 eyes (40.7%, 95% CI 39.2%-42.3%) had cataract or underwent cataract surgery. 883 people (44.0%, 95% CI 41.8%-46.2%) were diagnosed with cataract including 744 adults (37.1%, 95% CI 35.0%-39.2%) with binocular cataract and 139 adults (6.9%, 95% CI 5.9%-8.1%) with monocular cataract. 37 people (1.84%, 95% CI 1.3%-2.5%) underwent cataract surgery. The mean age of people took cataract surgery was 69±10.8 years old, higher than those who did not (59.9 ±9.4 years old). The prevalence of cortical, nuclear and PSC per person were 827 (41.4%, 95% CI 39.3–43.7), 607 (30.4%, 95% CI 28.4%-32.5%) and 29 (1.5%, 95% CI 1.0%-2.1%), respectively. The prevalence of cortical, nuclear and any cataract per person increased with age (Fig 1).

**Table 1:** Prevalence of Cataract and cataract surgery.

		Right Eye (%, 95% CI)	Left Eye (%, 95% CI)	Number of People (%, 95% CI)
**Any Cataract**		825 (41.3, 39.1–43.5)	802 (40.2, 38.0–42.3)	883 (44.0, 41.8–46.2)
	Cortical cataract	787 (39.8, 37.6–42.0)	752 (38.2, 36.0–40.4)	827 (41.4, 39.3–43.7)
	Nuclear cataract	555 (28.1, 26.1–30.1)	538(27.3, 25.4–29.3)	607 (30.4, 28.4–32.5)
	PSC	24 (1.2, 0.8–1.8)	20 (1.0, 0.6–1.6)	29 (1.5, 1.0–2.1)
	Cataract after surgery	21 (1.1, 0.7–1.6)	27 (1.4, 0.9–2.0)	37 (1.8, 1.3–2.5)
**No cataract**		1173 (58.7, 56.5–60.9)	1195 (59.9, 57.7–62.0)	1086 (54.1, 51.9–56.3)
**Total**		1998 (100)	1997 (100)	2006 (100)

PSC: posterior subcapsular cataract.

Table 2 showed the univariate and multivariable regression of risk factors for nuclear cataract. Using univariate analysis, age, shorter outdoor activity, longer cumulative UV-B exposure years, eyes with high myopia, eyes with AMD and other fundus diseases were risk factors of nuclear cataract. Using multivariable regression model, age (OR = 1.19, P<0.001), gender (OR = 1.56, P = 0.001) and high myopia (OR = 2.53, P = 0.038) were three independent risk factors of nuclear cataract.

**Table 2:** Univariate and multivariable association analysis of risk factors for nuclear cataract.

Risk factors		Univariate Analysis		Multivariate Analysis	
		P value	OR (95% CI)	P value	OR (95% CI)
**Age (y)**		<0.001***	1.190 (1.170–1.210)	<0.001***	1.190 (1.167–1.213)
**Gender**		0.190		0.001**	
	**Male**		1		1
	**Female**		1.140 (0.937–1.385)		1.559 (1.204–2.019)
**Outdoor activity (hours)**		<0.001***	0.928 (0.900–0.957)	0.646	1.010 (0.970–1.051)
**Cumulative UV-B exposure years (y)**		0.003**	1.012 (1.004–1.020)	0.654	1.002 (0.992–1.012)
**Sunlight Protection**		0.085		0.775	
	**Yes**		1.184 (0.977–1.434)		0.964 (0.750–1.240)
	**No**		1		1
**Glaucoma**		0.456		0.752	
	**Yes**		1.273 (0.675–2.403)		0.884 (0.412–1.898)
	**No**		1		1
**High myopia**		0.034*		0.038*	
	**Yes**		1.996 (1.055–3.773)		2.528 (1.055–6.062)
	**No**		1		1
**AMD**		<0.001***		0.084	
	**Yes**		4.393 (3.369–5.727)		1.335 (0.962–1.853)
	**No**		1		1
**Other fundus diseases**		<0.001***		0.251	
	**Yes**		3.300 (2.239–4.864)		1.364 (0.802–2.320)
	**No**		1		1

**AMD**: age-related macular degeneration.

*P<0.05

**P<0.01

***P<0.001.

Table 3 showed the univariate and multivariable analysis of risk factors for cortical cataract. Using univariate regression, age, gender, shorter outdoor activity, longer cumulative UV-B exposure years, eye protection, AMD and other fundus diseases were risk factors of cortical cataract. Using multivariable regression model, age (OR = 1.2, P<0.001), gender (OR = 1.86, P<0.001), outdoor activity (OR = 1.043, P = 0.03), cumulative UV-B exposure years (OR = 1.011, OR = 0.035) and other fundus diseases (OR = 2.04, P = 0.013) were independent risk factors of cortical cataract. The risk of cortical cataract increased 4.3% (95% CI 0.4%-8.3%) when outdoor activity time increased every one hour. Moreover, the risk of cortical cataract increased 1.1% (95% CI 0.1%-2.0%) when cumulative UV-B exposure time increased every one year.

**Table 3:** Univariate and multivariable association analysis of risk factors for cortical cataract.

Risk factors		Univariate Analysis		Multivariate Analysis	
		P value	OR (95% CI)	P value	OR (95% CI)
**Age (y)**		<0.001***	1.198 (1.178–1.218)	<0.001***	1.203 (1.181–1.226)
**Gender**		0.042*		<0.001***	
	**Male**		1		1
	**Female**		1.207 (1.007–1.448)		1.862 (1.456–2.380)
**Outdoor activity (hours)**		0.023*	0.968 (0.941–0.996)	0.029*	1.043 (1.004–1.083)
**Cumulative UV-B exposure years (y)**		<0.001***	1.024 (1.016–1.031)	0.035*	1.011 (1.001–1.020)
**Sunlight Protection**		0.041*		0.381	
	**Yes**		1.204 (1.007–1.440)		0.900 (0.710–1.140)
	**No**		1		1
**Glaucoma**		0.202		0.860	
	**Yes**		1.483 (0.810–2.716)		1.069 (0.508–2.252)
	**No**		1		1
**High myopia**		0.103		0.283	
	**Yes**		1.688 (0.900–3.168)		1.621 (0.671–3.914)
	**No**		1		1
**AMD**		<0.001***		0.245	
	**Yes**		4.317 (3.267–5.704)		1.223 (0.871–1.717)
	**No**		1		1
**Other fundus diseases**		<0.001***		0.013*	
	**Yes**		4.344 (2.821–6.691)		2.037 (1.160–3.574)
	**No**		1		1

**AMD**: age-related macular degeneration.

*P<0.05

**P<0.01

***P<0.001.

Table 4 showed the univariate and multivariable regression of risk factors for PSC. Using univariate analysis, age, outdoor activity and other fundus diseases were risk factors of PSC. Using multivariable regression model, only other fundus diseases (OR = 6.53, P<0.001) was independent risk factor of PSC.

**Table 4:** Univariate and multivariable association analysis of risk factors for posterior subcapsular cataract.

Risk factors		Univariate Analysis		Multivariate Analysis	
		P value	OR (95% CI)	P value	OR (95% CI)
**Age (y)**		0.041*	1.038 (1.002–1.076)	0.407	1.017 (0.977–1.060)
**Gender**		0.484		0.370	
	**Male**		1		1
	**Female**		1.317 (0.609–2.847)		1.462 (0.637–3.355)
**Outdoor activity (hours)**		0.038*	0.878 (0.776–0.993)	0.084	0.887 (0.774–1.016)
**Cumulative UV-B exposure years (y)**		0.124	0.975 (0.945–1.007)	0.086	0.970 (0.936–1.004)
**Sunlight Protection**		0.821		0.594	
	**Yes**		0.919 (0.441–1.914)		0.804 (0.361–1.793)
	**No**		1		1
**Glaucoma**		0.614		0.837	
	**Yes**		1.681 (0.223–12.682)		1.252 (0.147–10.641)
	**No**		1		1
**High myopia**		0.067		0.251	
	**Yes**		3.962 (0.907–17.313)		2.558 (0.515–12.704)
	**No**		1		1
**AMD**		0.936		0.744	
	**Yes**		1.045 (0.358–3.045)		0.826(0.263–2.593)
	**No**		1		1
**Other fundus diseases**		<0.001***		<0.001***	
	**Yes**		6.198 (2.561–15.004)		6.529 (2.512–16.970)
	**No**		1		1

**AMD**: age-related macular degeneration.

*P<0.05

**P<0.01

***P<0.001.

## Discussion

This study population was phrase 1 part summary of Taizhou Eye Study. Our study provided new population-based data on the association of outdoor activity and risk of cataract in rural residents aging ≥45 years old. We found that outdoor activity was risk factor for cortical cataract, but was not for nuclear and posterior subcapsular cataract. Furthermore, our study regarded outdoor activity density as a continuous variable and evaluated that the risk of cortical cataract increased 4.3% when outdoor activity time increased every one hour. Moreover, the risk of cortical cataract increased 1.1% (95% CI 0.1%-2.0%) when cumulative UV-B exposure time increased every one year.

Our study confirmed the cross-sectional association between outdoor activity and cortical cataract in Chinese population. Our result was consistence with some previous studies[20,21,31,32,33]. Epidemiology studies have showed that the prevalence rate of cataract in areas with low latitude and longer sunlight exposure is higher than areas with higher latitude and shorter sunlight exposure. In the U.S., the probability of cataract surgery increases 3% for every 1 degree decrease in latitude[34]. Latitude is directly associated with the UV-B degree of sunlight. UV-B is risk factor of cortical and nuclear cataract; ocular UV-B exposure may explain about 10% of the cortical cataract in the population[35]. The mechanisms of UV-B inducing cataract likely involve the apoptosis of human lens epithelium cells (HLECs). UV-B irradiation-initiated HLECs apoptosis may involve complicated mechanisms including mitochondrial dysfunction and caspase-3 activation[36]. The main dysfunction of mitochondrial was related to oxidative stress. Reduction of sunlight exposure is in many ways an effective means of preventing cataract related visual disability[35]. In our study, longer outdoor activity was also correlated with higher risk of cortical cataract (OR = 1.043, P = 0.03). Furthermore, the risk of cortical cataract increased 1.1% (95% CI 0.1%-2.0%) when cumulative UV-B exposure time increased every one year. Using the cumulative UV-B exposure year, it’s not surprising that some older individuals with few outdoor activity may have a higher risk of cortical cataract than the younger patient with more outside hours because the older individuals may have higher cumulative UV-B exposure years. However, we did not find the correlation of outdoor activity with nuclear cataract in our study, which might implicate different risk factors and prevention methods of different types of cataract in Chinese people.

Previous studies have showed that ARC occurs more common in female than male[3,37,38,39,40]. In our present study, female gender was also found as independent risk factors for cortical and nuclear cataract. The Blue Mountains Eye Study reported that the hormone replacement therapy could reduce the rate of cortical cataract and thus played a protective effect[41], the result was also proved in Salisbury Eye Evaluation[42]. However, contrary result was found in some other studies[43,44]. In a meta-analysis by Lai K. et al[45], Hormone therapy could significantly decrease the risk of nuclear cataract (OR = 0.72, 95% CI 0.61–0.85). Hormone replace therapy was not specifically studied in our study. However, prospective studies will be carried out in our future study about the association between hormone therapy and cataracts considering the limited numbers of studies in the meta-analysis.

High myopia has been proved as an independent risk factor of nuclear cataract in many epidemiologic studies[38,46,47]. In Beijing Eye Study[3], the age-adjusted nuclear cataract rate was significantly correlated with myopia. In the Beaver Dam Eye Study[46], the Los Angeles Latino Eye Study[38] and the Blue Mountains Eye Study[47], high myopia was also found to be associated with increased incidence of nuclear cataract. In our study, we also found high myopia (OR = 2.53, P = 0.038) as independent risk factor of nuclear cataract, which was consistent with previous studies. The potential mechanisms that high myopia leads to nuclear cataract are still unknown, but some researchers have hypothesized that axial myopia is a reason of a longer vitreous cavity that may cause decreased nutrition diffusion to the posterior lens and thus inhibits the oxidative defense mechanisms[38]. The hypothesis is supported by the fact that the hyperbaric oxygen treatment could result in a rapid nuclear cataract formation and that nuclear cataract occurs more common after vitrectomy surgeries, whereby the lens were exposed to a higher level of intraocular oxygen [48,49]. Prospective data from our study population may help to evaluate the direct role of myopia in the progression of nuclear cataract in our future work.

The potential risk factors of PSC include steroid usage, diabetic retinopathy, myopia and female gender in previous studies[11,19]. In our study, we only found the fundus diseases as a single strong independent risk factor for PSC (OR = 6.529, 95% CI 2.51–16.97). As the prevalence of PSC was very low (29 people, rate 1.4%) and the population was still limited in this study, we did not study the effect of diabetic retinopathy on PSC but put all of the other fundus diseases except AMD and glaucoma together. In our study, the effect of other fundus diseases on PSC was very strong, which implicated the significant role of fundus diseases on the development of PSC in Chinese population. However, specific fundus diseases will be studied in details of their effect on PSC in our further reports.

There were also some shortcomings of our manuscript; for example, we just evaluated the sunlight protection yes or no, instead of the accurate protection time. However, in this population-based epidemiologic study on the association of outdoor activity and different types of cataract in a Chinese rural population, we found that the outdoor activity was significantly associated with cortical cataract, but was not for nuclear and posterior subcapsular cataract. In conclusion, the risk of cortical cataract increased 4.3% when outdoor activity time increased every one hour. Furthermore, the risk of cortical cataract increased 1.1% when cumulative UV-B exposure time increased every one year. Our study gave an accurate quantitative evidence of the outdoor activity time effect on cortical cataract mechanisms and cataract prevention.

## References

[1] Brian G, Taylor H (2001) Cataract blindness—challenges for the 21st century. Bull World Health Organ 79: 249–256. 11285671 PMC2566371

[2] Editorial (2008) Blindness in the elderly. Lancet 372: 1273 10.1016/S0140-6736(08)61527-5 18929885

[3] Xu L, Cui T, Zhang S, Sun B, Zheng Y, Hu A, et al (2006) Prevalence and risk factors of lens opacities in urban and rural Chinese in Beijing. Ophthalmology 113: 747–755. 16650668 10.1016/j.ophtha.2006.01.026

[4] Zhou Q, Friedman DS, Lu H, Duan X, Liang Y, Yang X, et al (2007) The epidemiology of age-related eye diseases in Mainland China. Ophthalmic Epidemiol 14: 399–407. 18161614 10.1080/09286580701331974

[5] China national committee on aging office. (2014) China national committee on aging office. Prospects on China Elderly in the Next Century (Chinese). Available at: http://www.cnca.org.cn/include/content3.asp?thingid=10996.

[6] Zhang JS, Xu L, Wang YX, You QS, Wang JD, Jonas JB. (2011) Five-year incidence of age-related cataract and cataract surgery in the adult population of greater Beijing: the Beijing Eye Study. Ophthalmology 118: 711–718. 10.1016/j.ophtha.2010.08.021 21146222

[7] Huang W, Zheng Y, Wang L, Huang S, Liu B, Jin L, et al (2012) Five-year incidence and postoperative visual outcome of cataract surgery in urban southern China: the Liwan Eye Study. Invest Ophthalmol Vis Sci 53: 7936–7942. 10.1167/iovs.12-10903 23132796

[8] Cheng CY, Liu JH, Chen SJ, Lee FL (2000) Population-based study on prevalence and risk factors of age-related cataracts in Peitou, Taiwan. Zhonghua Yi Xue Za Zhi (Taipei) 63: 641–648. 10969451

[9] Li EY, Liu Y, Zhan X, Liang YB, Zhang X, Zheng C, et al (2013) Prevalence of blindness and outcomes of cataract surgery in Hainan Province in South China. Ophthalmology 120: 2176–2183. 10.1016/j.ophtha.2013.04.003 23714323

[10] Hu TS, Zhen Q, Sperduto RD, Zhao JL, Milton RC, Nakajima A. (1989) Age-related cataract in the Tibet Eye Study. Arch Ophthalmol 107: 666–669. 2719577 10.1001/archopht.1989.01070010684027

[11] Richter GM, Choudhury F, Torres M, Azen SP, Varma R (2012) Risk factors for incident cortical, nuclear, posterior subcapsular, and mixed lens opacities: the Los Angeles Latino eye study. Ophthalmology 119: 2040–2047. 10.1016/j.ophtha.2012.05.001 22771048 PMC3464350

[12] Klein BE, Klein R, Lee KE, Gangnon RE (2008) Incidence of age-related cataract over a 15-year interval the Beaver Dam Eye Study. Ophthalmology 115: 477–482. 10.1016/j.ophtha.2007.11.024 18171585

[13] Koo E, Chang JR, Agron E, Clemons TE, Sperduto RD, Ferris FR, et al (2013) Ten-year incidence rates of age-related cataract in the Age-Related Eye Disease Study (AREDS): AREDS report no. 33. Ophthalmic Epidemiol 20: 71–81. 10.3109/09286586.2012.759598 23510310

[14] Kanthan GL, Wang JJ, Rochtchina E, Tan AG, Lee A, Chia EM, et al (2008) Ten-year incidence of age-related cataract and cataract surgery in an older Australian population. The Blue Mountains Eye Study. Ophthalmology 115: 808–814. 17900695 10.1016/j.ophtha.2007.07.008

[15] Wu R, Wang JJ, Mitchell P, Lamoureux EL, Zheng Y, Rochtchina E, et al (2010) Smoking, socioeconomic factors, and age-related cataract: The Singapore Malay Eye study. Arch Ophthalmol 128: 1029–1035. 10.1001/archophthalmol.2010.147 20697004

[16] Richter GM, Chung J, Azen SP, Varma R (2009) Prevalence of visually significant cataract and factors associated with unmet need for cataract surgery: Los Angeles Latino Eye Study. Ophthalmology 116: 2327–2335. 10.1016/j.ophtha.2009.05.040 19815276 PMC2787839

[17] Delcourt C, Carriere I, Delage M, Descomps B, Cristol JP, Papoz L (2003) Associations of cataract with antioxidant enzymes and other risk factors: the French Age-Related Eye Diseases (POLA) Prospective Study. Ophthalmology 110: 2318–2326. 14644713 10.1016/s0161-6420(03)00713-9

[18] Navarro EJ, Gutierrez LJ, Valero CN, Buendia BJ, Calle PM, Martinez VV(2007) Prevalence and risk factors of lens opacities in the elderly in Cuenca, Spain. Eur J Ophthalmol 17: 29–37. 17294380 10.1177/112067210701700105

[19] Prokofyeva E, Wegener A, Zrenner E (2013) Cataract prevalence and prevention in Europe: a literature review. Acta Ophthalmol 91: 395–405. 10.1111/j.1755-3768.2012.02444.x 22715900

[20] Hirvela H, Luukinen H, Laatikainen L (1995) Prevalence and risk factors of lens opacities in the elderly in Finland. A population-based study. Ophthalmology 102: 108–117. 7831024 10.1016/s0161-6420(95)31072-x

[21] Delcourt C, Carriere I, Ponton-Sanchez A, Lacroux A, Covacho MJ, Papoz L (2000) Light exposure and the risk of cortical, nuclear, and posterior subcapsular cataracts: the Pathologies Oculaires Liees a l'Age (POLA) study. Arch Ophthalmol 118: 385–392. 10721962 10.1001/archopht.118.3.385

[22] Klein BE, Klein RE, Lee KE (1999) Incident cataract after a five-year interval and lifestyle factors: the Beaver Dam eye study. Ophthalmic Epidemiol 6: 247–255. 10544339 10.1076/opep.6.4.247.4190

[23] Tang Y, Wang X, Wang J, Huang W, Gao Y, Luo Y, et al (2015) Prevalence and Causes of Visual Impairment in a Chinese Adult Population: The Taizhou Eye Study. Ophthalmology. 10.1016/j.ophtha.2015.03.022 25986897

[24] Wang X, Lu M, Qian J, Yang Y, Li S, Lu D, et al (2009) Rationales, design and recruitment of the Taizhou Longitudinal Study. BMC Public Health 9: 223 10.1186/1471-2458-9-223 19589173 PMC2715397

[25] Zhao J, Ellwein LB, Cui H, Ge J, Guan H, Lv J, et al (2010) Prevalence of vision impairment in older adults in rural China: the China Nine-Province Survey. Ophthalmology 117: 409–416, 411–416 10.1016/j.ophtha.2009.11.023 20079923 PMC6029941

[26] Chylack LJ, Wolfe JK, Singer DM, Leske MC, Bullimore MA, Bailey IL, et al (1993) The Lens Opacities Classification System III. The Longitudinal Study of Cataract Study Group. Arch Ophthalmol 111: 831–836. 8512486 10.1001/archopht.1993.01090060119035

[27] Liang YB, Friedman DS, Wong TY, Zhan SY, Sun LP, Wang JJ, et al (2008) Prevalence and causes of low vision and blindness in a rural chinese adult population: the Handan Eye Study. Ophthalmology 115: 1965–1972. 10.1016/j.ophtha.2008.05.030 18684506

[28] Klein R, Davis MD, Magli YL, Segal P, Klein BE, Hubbard L (1991) The Wisconsin age-related maculopathy grading system. Ophthalmology 98: 1128–1134. 1843453 10.1016/s0161-6420(91)32186-9

[29] Foster PJ, Buhrmann R, Quigley HA, Johnson GJ (2002) The definition and classification of glaucoma in prevalence surveys. Br J Ophthalmol 86: 238–242. 11815354 10.1136/bjo.86.2.238 PMC1771026

[30] McCarty CA, Lee SE, Livingston PM, Bissinella M, Taylor HR (1996) Ocular exposure to UV-B in sunlight: the Melbourne visual impairment project model. Bull World Health Organ 74: 353–360. 8823956 PMC2486882

[31] Sasaki H, Kawakami Y, Ono M, Jonasson F, Shui YB, Cheng HM, et al (2003) Localization of cortical cataract in subjects of diverse races and latitude. Invest Ophthalmol Vis Sci 44: 4210–4214. 14507863 10.1167/iovs.01-1221

[32] Cruickshanks KJ, Klein BE, Klein R (1992) Ultraviolet light exposure and lens opacities: the Beaver Dam Eye Study. Am J Public Health 82: 1658–1662. 1456342 10.2105/ajph.82.12.1658 PMC1694542

[33] Age-Related Eye Disease Study Research Group (2001) Risk factors associated with age-related nuclear and cortical cataract: a case-control study in the Age-Related Eye Disease Study, AREDS Report No. 5. Ophthalmology 108: 1400–1408. 11470690 10.1016/s0161-6420(01)00626-1 PMC1473213

[34] Javitt JC, Taylor HR (1994) Cataract and latitude. Doc Ophthalmol 88: 307–325. 7634999 10.1007/BF01203684

[35] Congdon NG (2001) Prevention strategies for age related cataract: present limitations and future possibilities. Br J Ophthalmol 85: 516–520. 11316704 10.1136/bjo.85.5.516 PMC1723947

[36] Ji Y, Cai L, Zheng T, Ye H, Rong X, Rao J, et al (2014) The mechanism of UVB irradiation induced-apoptosis in cataract. Mol Cell Biochem. 10.1007/s11010-014-2294-x 25445170

[37] Tsai SY, Hsu WM, Cheng CY, Liu JH, Chou P (2003) Epidemiologic study of age-related cataracts among an elderly Chinese population in Shih-Pai, Taiwan. Ophthalmology 110: 1089–1095. 12799231 10.1016/S0161-6420(03)00243-4

[38] Richter GM, Torres M, Choudhury F, Azen SP, Varma R (2012) Risk factors for cortical, nuclear, posterior subcapsular, and mixed lens opacities: the Los Angeles Latino Eye Study. Ophthalmology 119: 547–554. 10.1016/j.ophtha.2011.09.005 22197433 PMC3293944

[39] Mahdi AM, Rabiu M, Gilbert C, Sivasubramaniam S, Murthy GV, Ezelum C, et al (2014) Prevalence and risk factors for lens opacities in Nigeria: results of the national blindness and low vision survey. Invest Ophthalmol Vis Sci 55: 2642–2651. 10.1167/iovs.12-10303 24526441

[40] Na KS, Park YG, Han K, Mok JW, Joo CK (2014) Prevalence of and risk factors for age-related and anterior polar cataracts in a Korean population. PLoS One 9: e96461 10.1371/journal.pone.0096461 24936893 PMC4060994

[41] Younan C, Mitchell P, Cumming RG, Panchapakesan J, Rochtchina E, Hales AM (2002) Hormone replacement therapy, reproductive factors, and the incidence of cataract and cataract surgery: the Blue Mountains Eye Study. Am J Epidemiol 155: 997–1006. 12034578 10.1093/aje/155.11.997

[42] Freeman EE, Munoz B, Schein OD, West SK (2001) Hormone replacement therapy and lens opacities: the Salisbury Eye Evaluation project. Arch Ophthalmol 119: 1687–1692. 11709021 10.1001/archopht.119.11.1687

[43] Lindblad BE, Hakansson N, Philipson B, Wolk A (2010) Hormone replacement therapy in relation to risk of cataract extraction: a prospective study of women. Ophthalmology 117: 424–430. 10.1016/j.ophtha.2009.07.046 20045566

[44] Klein BE, Klein R, Lee KE (2000) Reproductive exposures, incident age-related cataracts, and age-related maculopathy in women: the beaver dam eye study. Am J Ophthalmol 130: 322–326. 11020411 10.1016/s0002-9394(00)00474-8

[45] Lai K, Cui J, Ni S, Zhang Y, He J, Yao K. (2013) The effects of postmenopausal hormone use on cataract: a meta-analysis. PLoS One 8: e78647 10.1371/journal.pone.0078647 24205286 PMC3813478

[46] Wong TY, Klein BE, Klein R, Tomany SC, Lee KE (2001) Refractive errors and incident cataracts: the Beaver Dam Eye Study. Invest Ophthalmol Vis Sci 42: 1449–1454. 11381046

[47] Kanthan GL, Mitchell P, Rochtchina E, Cumming RG, Wang JJ (2014) Myopia and the long-term incidence of cataract and cataract surgery: the Blue Mountains Eye Study. Clin Experiment Ophthalmol 42: 347–353. 10.1111/ceo.12206 24024555

[48] Palmquist BM, Philipson B, Barr PO (1984) Nuclear cataract and myopia during hyperbaric oxygen therapy. Br J Ophthalmol 68: 113–117. 6691953 10.1136/bjo.68.2.113 PMC1040267

[49] Holekamp NM, Shui YB, Beebe DC (2005) Vitrectomy surgery increases oxygen exposure to the lens: a possible mechanism for nuclear cataract formation. Am J Ophthalmol 139: 302–310. 15733992 10.1016/j.ajo.2004.09.046

